# Fast and Accurate Object Detection in Remote Sensing Images Based on Lightweight Deep Neural Network

**DOI:** 10.3390/s21165460

**Published:** 2021-08-13

**Authors:** Lei Lang, Ke Xu, Qian Zhang, Dong Wang

**Affiliations:** School of Computer and Information Technology, Beijing Jiaotong University, Beijing 100044, China; leilang@mails.ccnu.edu.cn (L.L.); 17112071@bjtu.edu.cn (K.X.); 19140123@bjtu.edu.cn (Q.Z.)

**Keywords:** remote sensing image, object detection, anchor configurations, differential evolution, YOLO, attention module

## Abstract

Deep learning-based object detection in remote sensing images is an important yet challenging task due to a series of difficulties, such as complex geometry scene, dense target quantity, and large variant in object distributions and scales. Moreover, algorithm designers also have to make a trade-off between model’s complexity and accuracy to meet the real-world deployment requirements. To deal with these challenges, we proposed a lightweight YOLO-like object detector with the ability to detect objects in remote sensing images with high speed and high accuracy. The detector is constructed with efficient channel attention layers to improve the channel information sensitivity. Differential evolution was also developed to automatically find the optimal anchor configurations to address issue of large variant in object scales. Comprehensive experiment results show that the proposed network outperforms state-of-the-art lightweight models by 5.13% and 3.58% in accuracy on the RSOD and DIOR dataset, respectively. The deployed model on an NVIDIA Jetson Xavier NX embedded board can achieve a detection speed of 58 FPS with less than 10W power consumption, which makes the proposed detector very suitable for low-cost low-power remote sensing application scenarios.

## 1. Introduction

With the rapid development of satellite and imaging technology, optical remote sensing images with high spatial resolution are obtained more conveniently than ever before [1]. Studies on analyzing and understanding remote sensing images have drawn wide attention. Image classification, segmentation, object detection, and tracking have become the hot topics in the field of remote sensing [2,3,4]. Among them, object detection has presented a broader application prospect in real-world applications and is sought after by researchers in recent years [5].

For object detection tasks, deep neural network-based schemes have shown superior performance over traditional approaches [6,7]. In general, these schemes can be divided into two major categories: (1) one-stage neural network which adopts a fully convolutional architecture that outputs a fixed number of predictions on the grid, such as SSD [8], YOLO [9], and M2Det [10], and (2) two-stage network that leverages a proposal network to find regions of interest that have a high probability to contain an object and a second network to get the classification score and spatial offsets, such as FPN [11] and Faster R-CNN [12]. These detectors have been successfully utilized in many applications, such as robotics, autonomous vehicles, and surveillance systems.

However, a direct utilization of generic detectors in remote sensing images usually does not deliver satisfactory results. The major reason is that there are many distinct features different from natural images in remote sensing images, such as very complex geometric background, dense object distributions, and variety of objects with large variant in shapes and scales [13,14]. To address these new design challenges faced in object detection in remote sensing images, many studies have been proposed in the literature [15,16,17,18,19,20]. For instance, Wang et al. [15] designed a new pyramid structure to optimize the Faster-RCNN detector by adding a feature-reflowing pathway from the lower level for each scale to enrich the feature expression. Huang et al. [16] proposed a cross-scale fusion module based on the M2Det detector to extract sufficient comprehensive semantic information from features for performing multi-scale fusion. Zhao et al. [17] improved the SSD detector by adding a channel attention module to strengthen the long-term semantic dependence between objects to improve the discriminative ability of the deep features.

**Figure 1:**
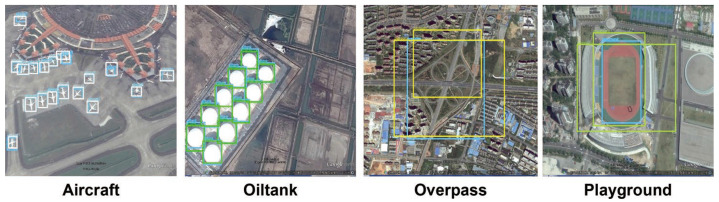
Demonstration of complicated remote sensing scenes in RSOD dataset. The detected bounding box are draw by using YOLOv4-Tiny.

In this paper, we focus on the issue of the large variant in object distributions and scales in remote sensing images. Traditional anchor-based detectors [21,22] match regions of possible objects by a set of pre-allocated anchors with pre-defined aspect ratios; therefore, the final accuracy of the trained neural network model highly relies on the anchor configuration. However, in remote sensing images, the scale and distribution of the target objects vary in a very wide range. For instance, Figure 1 compares the sizes of the four classes of objects in the RSOD [23] dataset. Due to the fact that remote sensing images are often acquired by sensors with the same hardware settings, and because the shooting angle is also fixed [24], the sizes of the target object in remote sensing images are directly related to the real-world object scale. As a result, there is a large gap in the size of the ground truth bounding boxes among all objects within one training dataset. In Figure 1, the aircraft and oiltanks have much smaller scales but larger quantities in the image when compared to the overpass and playground. The current anchor selection scheme, such as K-means clustering [25,26,27], tends to allocate more anchors for objects with larger quantities. This makes generic detector perform very badly on objects that does not have sufficient corresponding samples during anchor configuration. To demonstrate this phenomenon, we compare some representative detection results of using the YOLOv4-Tiny network on the RSOD dataset in Figure 1. For the object classes of overpass and playground, the detection results are very inaccurate.

Beside the above problems, this study also deals with the efficiency of the deep neural network which consists most parts of the detector. In real-world applications, remote sensing object detection tasks are commonly used in rescue, military, and other scenarios [28,29]. This requires the detectors to be as lightweight as possible and be able to efficiently deploy on low-cost low-power embedded devices. Regardless of the type of detection framework used, optimizations of the detailed algorithm, such as the backbone neural network, multi-scale feature fusion, and adaptive anchor setting, are also important in trading off between the detection accuracy and speed to meet the requirements of the target application.

The contributions of this paper are the following. (1) We propose a lightweight backbone deep neural network design, which can achieve the optimal balance between model size and detection accuracy for fast processing on low-cost low-power embedded hardware platforms. (2) We propose an automatic anchor configuration scheme based on differential evolution (DE), which can minimize the average distance between ground truth bounding boxes and selected anchors, and improve the accuracy of object matching. (3) Comprehensive experiments on multiple datasets are conducted, and the results show that the proposed lightweight detector outperforms state-of-the-art detection approaches by 5.13% and 3.58% on the RSOD and DIOR datasets, respectively. We have also deployed the proposed detector on a embedded hardware platform, i.e., an NVIDIA Jetson Xavier NX board [30], achieving a real-time detection speed of 58 FPS with less than 10 W power consumption.

The reminder of this paper is organized as follows. The related work on existing studies of deep neural network design for remote sensing object detection tasks is summarized in Section 2. The proposed neural network design and automatic anchor configuration scheme is introduced in Section 3. The experimental setup is shown in Section 4. Section 5 describes the experimental results in detail, whereas Section 6 shows the performance of the deployment on embedded platform. Finally, Section 7 concludes this paper.

## 2. Related Work

The identification of objects in remote sensing images is a subset of a wider research field in object detection. Taking into account the needs of neural network deployment, the existing studies can be categorized into three main groups: lightweight object detection network, visual attention mechanism, and optimized anchor configuration.

### 2.1. Lightweight Object Detection Network

State-of-the-art approaches can be divided into region-based and single-shot detectors. The former ones, such as R-CNN [31], Fast R-CNN [32], and Faster R-CNN, use a set of Regions-of-Interest (ROIs) to extract sub-promotions that may contain objects, then fine-grained detection and classification modules analyze each ROI. Although region-based schemes have high detection accuracy, the complex network structures often result in high computation workload and low processing speed. Single-shot detectors, such as SSD, YOLO, and FCOS [33], tackle the input image in a single pipeline. As a benefit of the end-to-end structure, these detectors can achieve real-time detection speed, which is very suitable as a lightweight object detection network structure. Designers can further balance the accuracy and model size according to the requirements of their own target application.

As the most typical end-to-end network structure, YOLO [9,27,34,35] is favored by many application scenarios [36,37,38]. YOLO-Tiny is the lightweight version of the YOLO detector. YOLO detector is generally composed of three basic parts: backbone, neck, and head [35]. The backbone neural network, as an important part of the network to extract deep image information, generally performs as the image classification network, such as Darknet53, ResNet [39], Vgg [40], and Mobilenet [41]. The key information of object is extracted in the neck, and used to predict the position and category in the head. Although YOLO-Tiny meets the actual deployment requirements, it could not meet the accuracy requirements on remote sensing images due to the following drawbacks:

- The shallow backbone and prediction network structures are not sufficient to extract deep semantic information, which limits the performance of the network in complex scenarios, for instance, very small objects or complicated backgrounds [42]. In addition, the simple organization of prediction layers cannot effectively cover objects of various proportions, especially when remote sensing images with dense object distributions were considered.
- The performance of the detector is especially sensitive to anchor configurations, which not only affects the speed of training, but also the robustness of the network. As been explained in previous section, the small amount of anchors with fixed scales in YOLO-Tiny will deliver poor detection results due to the large variation in object scales.

### 2.2. Visual Attention Mechanism

Aiming at highlighting the salient feature of images, the attention module has been widely used in various types of detectors. It has became the mainstream approach of improving network accuracy. For instance, Wang et al. [43] proposed the residual attention network, which improved the expressive ability of the network. A channel-level attention mechanism through feature recalibrating was proposed by SENet [44], which could improve neural network’s performance in classification, detection, and other tasks to a new extent. CBAM [45], which combined channel attention and spatial attention, was also proposed to further improve the model’s accuracy. The CA module [46] that integrated the channel and the spatial direction not only reduced the amount of parameters, but also had stronger capabilities. In general, a typical attention module can be divided into two parts: channel attention module and spatial attention module, which have their own advantages and disadvantages for different applications, and the specific effects were often evaluated based on experiments on the target dataset.

Although the attention module can deliver increased detection accuracy, the improvements often come from objects of large scales, which means that, due to the special calculation utilized, some unobvious features are ignored, and the attention model usually focuses on feature-rich large objects, while small objects [46] are often ignored. For remote sensing images with a large number of small objects, the effectiveness of attention modules still needs to be further investigated.

### 2.3. Optimized Anchors

Anchor is used by the YOLO framework in the detection pipeline, which is extremely important in remote sensing image-based object detection tasks. In previous studies, there are two commonly used methods to allocate the anchors for the detection network:

1. Manual configuration. The anchors selected by the manual correction method are more straightforward and robust. However, it requires the designer to have rich experience in the application field and perform comprehensive manual experiments before determining the best setting.
2. Automatic configuration based on optimizations. According to distribution of the data set, this type of scheme can automatically find the best anchor position, which greatly relieves the effort of searching for the optimal configurations and also delivers higher accuracy and faster training speed.

K-means clustering is widely used as the optimization scheme for automatic anchor selection in YOLO. Given a specific number of anchors, the best anchor positions are selected by clustering the bounding boxes in the datasets. The IOU score is further used as the relative distance for anchor configurations in YOLOv4 [35]. Junos et al. [47] used a similar scheme to optimize the anchor in YOLOv3 and applied the network to the crop harvesting system. Zlocha et al. [48] optimized the anchor configuration based on a differential evolution search algorithm in RetinaNet. However, in previous studies, all objects were unified into one big category, which was not reasonable for datasets with unevenly distributed object scales. As we have pointed out in the introduction section, traditional scheme tends to shift the anchor selection toward the category with the maximum number of ground truth samples, resulting in poor detection performance for other objects.

## 3. Methodology

**Figure 2:**
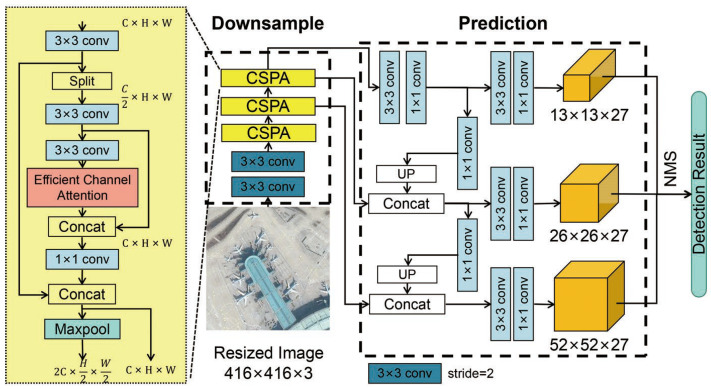
The proposed lightweight neural network for remote sensing object detection.

In this paper, we aim to design a lightweight deep neural network to accurately detect objects of large variant in scale and quantity in remote sensing images. The proposed detector framework is illustrated in Figure 2. In the proposed detector, we have designed a YOLOv4-like backbone network followed by three prediction layers to capture and combine rich contextual features, while, at the same time, minimizing the network’s computational cost. Moreover, efficient channel attention is also developed to form a downsampling module, namely, the Cross Stage Partial connections with Attention (CSPA), to efficiently extract high-level feature information for object classification and localization. Finally, an automatic optimal anchor selection approach based on differential evolution (DE) is proposed to address the problem of biased anchor allocation due to large variant of object scale and quantity in remote sensing images.

### 3.1. Lightweight Neural Network

In this section, the details of the proposed neural network architecture are presented. Our network design emphasizes the lightweight feature and computational efficiency at the minimum cost of detection accuracy. The overall neural network architecture is illustrated in Figure 2.

The proposed network is organized in two major components: a downsampling backbone and a prediction pipeline. The input images are first resized to an appropriate size (for instance, 416 × 416 pixels), and then the downsampling backbone network, which has a YOLOv4-like architecture consisting of two convolutional layers and three CSPA modules, extract the salient features form the input image at different layers. To enhance the network’s ability of capturing important features within one channel and fusing information among different input channels, we propose to add an efficient channel attention layer after the third convolution layer in each of the CSPA module.

In the proposed network, a prediction pipeline consisting of feature pyramids and three-scale prediction layers is designed to transform features from multiple branches into prediction results. The feature pyramid can effectively fuse meaningful semantic information obtained from salient feature maps of low resolution and finer-grained information extracted from the earlier branches. The three-scale prediction networks are used to predict encoding parameters of bounding box and class predictions. The final inference results are obtained after filtering the prediction box via non-maximal suppression (NMS).

**Figure 3:**
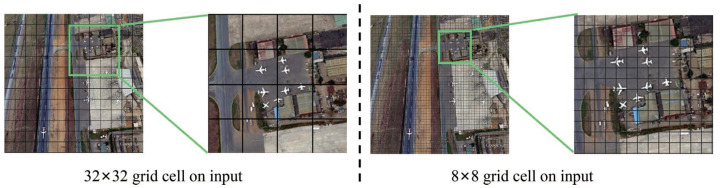
Different grid cells on input.

In the original YOLOv4-Tiny structure, only two layers of networks were utilized in the prediction part to alleviate the computational complexity of the excessively large network in YOLOv4. However, this shallow network structure is extremely unfriendly to remote sensing object detection task. For instance, given an input image of the resolution of 416 × 416 pixels, the dimensions of the output feature maps of the second prediction layer in the original YOLOv4-Tiny structure are 26 × 26. According to the principle of the YOLO algorithm, the input image is divided into many grid cells of equal dimension, each of which will detect objects that appear within it. In the case of using two prediction layers, the image will be divided into 26 × 26 grid cells, which means that each cell is of the size 16 × 16. Generally, when the scales of the target object are too small relative to the size of the grid cells, the object could not be precisely located by the YOLO algorithm. In remote sensing images, there exists a large amount of small objects which cannot be precisely located by grid cells larger than 16 × 16. Therefore, in this work, we propose to add one extra prediction layer with an output feature map dimensions of 56 × 56, which corresponds to grid cells of 8 × 8 dimension on the input image. From Figure 3, it can be seen that, in the proposed network, the corresponding grid cells mapped on the input images well fit the scales of the small objects. Moreover, based on quantitative analysis (please refer to the detailed result on RSOD in Section 5.1), we have found that the proposed three-layer prediction network was optimal and there was no need to introduce extra layers at the expense of larger model size for very little improvement in detection accuracy.

### 3.2. Efficient Channel Attention

**Figure 4:**
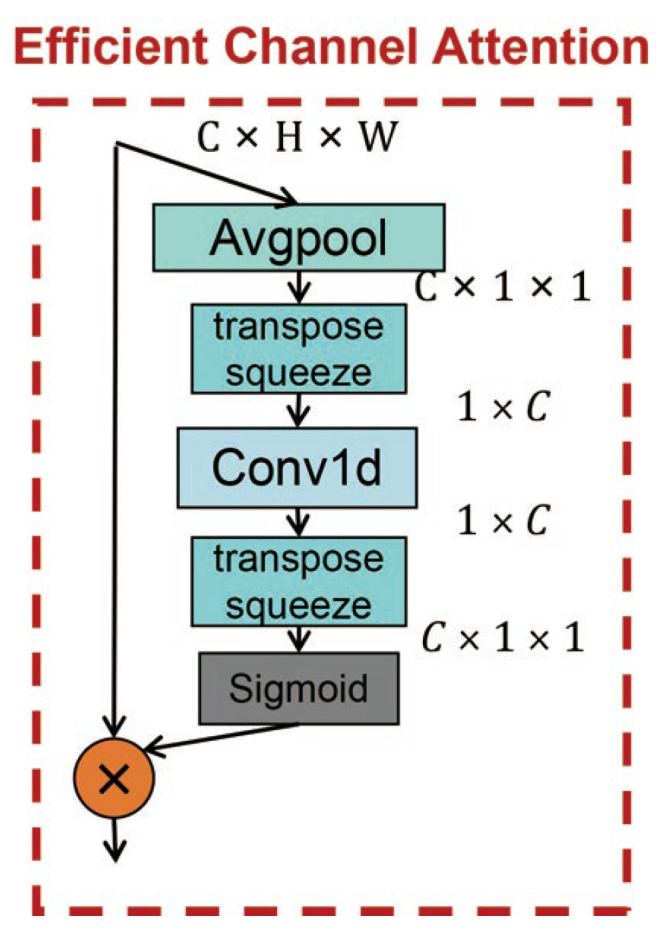
Structure of ECA module.

Instead of utilizing both channel and spatial level attentions, we propose to use a single channel attention module to enhance the visual attention of the backbone network. It is based on two design considerations: First, channel attention modules normally have a relatively small amount of parameters compared to spatial ones, which can facilitate rapid training convergence and deliver a faster prediction speed during deployment. Second, we have conducted experiments showing that using channel attention modules was sufficient to improve the detection accuracy to the desired level for remote sensing images. In this work, an efficient channel attention module was designed and utilized in the backbone network by following the basic network structure presented in [49] as shown by Figure 4.

The detailed architecture of the Efficient Channel Attention (ECA) module is summarized as follows: Given the input feature map *I*, $I\in R^{C\times W\times H}$. In the first step, an average pooling operation is designed to compress *I* along the spatial dimension W×H to obtain the channel descriptor (${\mathbb{R}}^{C\times 1\times 1}$). In the following layer, it is converted into another channel descriptor ($R^{1\times C}$) through transpose and squeeze operations. Then, the local cross-channel interaction is completed through fast 1D convolution of the kernel size $k_{size}$, where $k_{size}$ also represents the coverage ratio of interaction. In the following parts, transpose and squeeze operation are utilized again to convert channel descriptor back to the original channel dimensions ($R^{C\times 1\times 1}$). Finally, the channel weight is generated through the Sigmoid function, and multiplied by *I*.

To compare the performance of the classic channel attention module (Squeeze-and-Excitation (SE) [44]) with the proposed ECA module in terms of feature extraction capability and operation efficiency, we have conducted experiment to quantitatively measure the increment in model size and improvement in detection accuracy for both schemes when adopted in the proposed backbone network. The results are reported in Table 1. It can be observed that the SE module outperforms the baseline model by 3.10%, but with an increment of 2688 parameters, while ECA module outperforms SE module by 0.22% but contributes to 74% less parameters. It can be concluded that the ECA module can capture sufficient cross-channel interaction in an efficient way to improve the detection performance with minimal cost in model size and computational complexity.

### 3.3. Optimal Anchor Configuration Based on Differential Evolution

The anchor configuration, a hyperparameter for the training of the network model, affects the performance of the model in a great degree. Optimal anchor configuration can improve the network accuracy without additional consumption. However, when facing the large variation of object scales in remote sensing images, the anchor configuration scheme used in YOLO, i.e., K-means clustering, will lead to biased anchor allocating setting. The improvement in accuracy tends to be concentrated in the categories with a larger number of objects. One major reason is that the key evaluation metric widely used by previous studies is the mAP score, which is embodied as an average AP score of all categories. Excessively increasing the AP score of a certain category can increase the overall accuracy of the model to a certain extent. However, further improvement is not possible due to the limited detection ability for the other categories with very low AP scores, which means that the trained model is biased. In general, a more reasonable approach is to consider both the distribution of the quantity and size of the objects for all categories, and develop a scheme that can improve the overall performance of the neural network and balance the accuracy of all target categories.

To better capture the relationship between object scale and quantity, we propose an improved anchor configuration scheme based on differential evolution (DE). This method takes the height and width of the anchors as variables, and the sum of the nearest distances from the ground truth bounding boxes to the anchors as a fitness function. In addition, a weight value is also added to the distance calculation to avoid possible biased training of the neural network. Finally, the minimum value of the fitness function is solved by using DE [50], reaching the goal of minimizing the distance.

More specifically, for a given dataset, the distance from one ground truth bounding box to one anchor can be formulated as follows:

(1) dis(truth,anchor)=1−IOU(truth,anchor)

where IOU(truth,anchor) represents the intersection over union of the ground truth and the anchor box, centered at the origin. The corresponding calculation formula is

(2) $$IOU=\frac{S_{overlap}}{S_{union}}$$

where $S_{overlap}$ refers to the overlap area between the ground truth and the anchor box. $S_{union}$ refers to the union area between them.

By denoting $x_{ij}$ as the *j*-th ground truth of the *i*-th category, and $\theta_{k}$ as *k*-th anchor box, the distance between $x_{ij}$ and the anchor boxes can be expressed in the following form:

(3) $$g\left(x_{ij},\theta \right)=min\left(dis\left(x_{ij},\theta_{1}\right),dis\left(x_{ij},\theta_{2}\right),\cdots ,dis\left(x_{ij},\theta_{k}\right)\right)$$

which means that we choose the anchor with the smallest distance as its best match. Therefore, the distance between all samples (*X*) and anchors (θ) can be calculated by

(4) $$G=\frac{1}{n}\times \sum_{i=1}^{n}\left[\frac{1}{m_{i}}\times \sum_{j=1}^{m_{i}}g\left(x_{ij},\theta \right)\right]$$

where $m_{1}$, $m_{2}$, …, $m_{n}$ represent the number of ground truth samples in the *n*-th category.

In Equation (4), the values of the weights for different categories in the fitness function are set to be inversely proportional to the number of objects, which helps to eliminate the attraction effect of large number of objects for more anchors.

Here, we use single-objective DE [51] to solve this optimization problem of Equation (4). In Equation (4), the real values in $x_{ij}$ and $\theta_{k}$ are all scaled to the range of [0,1]. To prevent conflicting with the aforementioned variables, the decision variables in DE are replaced by $P_{i}$, i.e., $P_{i}=\theta \in \left(k,2\right)$ used as the fitness function of DE. Then, DE searches for the best anchors of the smallest function *G* through constant iterations. The pseudocode of the proposed optimal anchor selection scheme is described by Algorithm 1, where $C_{r}$ denotes crossover rate, $F_{s}$ represents the scaling factor, $N_{p}$ is the population size, and *t* is the iteration number.
**Algorithm 1** Anchor configurations algorithm based on DE.**Input:** input parameters $C_{r}$, $F_{s}$, $N_{p}$**Output:** output $argmin_{P_{i}^{t}}G\left(P_{i}^{t}\right)$ and $P_{i}^{t}$
1:Initialize population $P=(P_{1}^{t},P_{2}^{t},\cdots ,P_{N_{p}}^{t})$2:Counter t←03:**while** stop condition not met **do**4:    **for** $i\in (1,2,\cdots ,N_{p})$ **do**5:        $\nu_{i}\leftarrow$differential mutation $\left(F_{s};i,P\right)$6:        $\mu_{i}\leftarrow$crossover $\left(C_{r};P_{i}^{t},\nu_{i}\right)$7:        **if** $G\left(\mu_{i}\right)\geq G\left(P_{i}^{t}\right)$ **then**8:           $P_{i}^{t+1}\leftarrow \mu_{i}$9:        **else**10:           $P_{i}^{t+1}\leftarrow P_{i}^{t}$11:        **end if**12:    **end for** t←t+113:**end while**14:**return**$argmin_{P_{i}^{t}}G\left(P_{i}^{t}\right)$ and $P_{i}^{t}$

In the first step, the proposed algorithm performs an initialization operation on the population *P* consisting of decision variables $P_{i}^{t}$, where $P_{i}^{t}$ represents the *i*-th individual in the *t*-th iteration. The initialized population is distributed in a certain defined area according to the population size $N_{p}$, and each individual represents a candidate solution. Generally, the initial population should cover the whole search space. As the population size $N_{p}$ increases, the probability of obtaining the global optimal solution also increases. In this work, each individual is defined as a specific anchor configuration.

In the following iteration, the procedures of differential mutation, crossover, and selection are repeated performed. The goal of differential mutation is to create groups of new individuals which have a certain level of probability of being the optimal solution. The difference introduced by mutant between the parent and children is quantitatively controlled by a scaling factor $F_{s}$. Later, elements $\theta_{j},(j=1,2,\cdots ,k)$ in individuals are randomly swapped by the crossover operation for the current iteration and its differential mutated group. This procedure promotes population diversity, and the crossover probability is controlled by $C_{r}$. Then, newly generated and contemporary individuals are compared and better individuals are selected for transmission to the next iteration. The iteration continues until the best individual and objective function value are found. Through the above algorithm, the minimum value of fitness function can be obtained, which corresponds to the best anchor value.

## 4. Experimental Settings

### 4.1. Hardware Platforms

The proposed lightweight model was expected to run on low-power embedded devices. However, to demonstrate the advantage of the proposed optimization schemes and the performance of the proposed detector more comprehensively, experiments were conducted on two different hardware platforms, including an NVIDIA GeForce RTX2080Ti desktop GPU and an NVIDIA Jeteson Xavier embedded board. The hardware specifications of the two platforms are summarized in Table 2. To deploy on the embedded board, all tested neural network model were quantized into FP16 data format to relieve the pressure on external memory bandwidth.

### 4.2. Datasets and Training Parameters

In this work, experiments on two public remote sensing datasets were conducted to verify the effectiveness of the proposed detector and further evaluate its accuracy and speed. The RSOD remote sensing dataset was selected to measure the key performance metrics of the detector. RSOD includes 4993 aircraft in 446 images, 1586 oiltanks in 165 images, 191 playgrounds in 189 images, and 180 overpasses in 176 images. The dataset was randomly divided into the training and test set according to a 7.5:2.5 ratio.

Considering the relatively limited number of samples in the RSOD, in order to further verify the performance of our method, we have also chosen DIOR to evaluate and test the performance of the proposed model. DIOR [52] contains a wider range of 20 object categories, a total number of 23,463 images, in which 192,472 examples were labeled, and 11,725 images were used for training and 11,738 images were used for testing.

The training parameters of the final network model was setting as follows: The initial weight was pre-trained on the COCO dataset; Adam was utilized as the optimizer, while the initial learning rate was $1\times 10^{-4}$ and the maximum training epoch was set to 100.

### 4.3. Evaluation Metrics

We used a total number of four metrics to evaluate the performance of the proposed method: (1) mAP; (2) FLOPs (floating-point operations); (3) number of parameters; (4) FPS (frames per second). The mean average precision (mAP), which is widely used by previous studies, is still used as the key accuracy score in this paper to compare with the state-of-the-art. FLOPs and parameters are used to evaluate the computational complexity and memory footprint of the neural network model, respectively. FPS is used to evaluate the processing speed of the model on target hardware devices.

## 5. Results

### 5.1. Improvements by Network Structure

In this paper, we have proposed two optimizations on the lightweight neural network: multi-scale prediction layers and attention modules. The performance gains of these two schemes were evaluated independently and the results are shown in the following sections.

As discussed in Section 3.1, a deeper prediction network corresponds to a finer grid on the input images, which can locate small objects more accurately. However, detection speed will often be sacrificed due to increased model size and computational complexity. To balance between processing speed and accuracy, we have conducted a series of experiments to explore the optimal structure of the prediction network. The obtained results are compared in Table 3. Compared to only using two prediction layers, adopting a three layer prediction network can improve the detection accuracy in terms of mAP score by 2%, while the increment in FLOPs is ~48%. In particular, the detection accuracy of overpass and aircraft, which often have smaller scales in the images, are improved by 2.27% and 7.74%, respectively. Although adding a fourth layer to the prediction network can further raise the average accuracy by around 1% (the improvement is mainly on the small object of aircraft), the computational complexity doubled compared to using three layers and is 3× than that of using only two layers. In addition, the extra prediction layer also creates a large amount of unnecessary bounding boxes, which will also reduce the executing speed of the post-processing procedures, such as NMS. Therefore, it is concluded, based on the comparative experiment results, that the three layer prediction network architecture was the most cost-effective design.

For visual attention enhancement, the proposed ECA design was compared with several state-of-the-art attention modules, including SE, CBAM, and CA, as listed in Table 4. The parameter count only includes the parameters introduced by the attention module, while the other metrics correspond to the whole neural network. The proposed ECA module delivers a 3.32% boost in mAP with almost no loss in processing speed. Although the CBAM and CA modules have both channel and spatial attention, they failed to improve the detection results. The main reason behind this was that these two attention modules that were added at a position close to the input have introduced a larger amount of parameters (4×thanthatoftheproposedscheme) to the original model, causing the training process very sensitive to the initialization state of the backbone model and the original loss function failed to generate sufficient backpropagation information to update the new parameters. The SE module has a very close inference performance with the proposed ECA module, but the processing speed is 2% slower on desktop GPU. This cost in speed will be further enlarged when deployed on embedded platforms.

The proposed ECA module also has a hyperparameter $k_{size}$, i.e., the filter size of the 1D convolution. Table 5 shows the experimental results with different values of $k_{size}$, including setting fixed value and adaptive ones in all convolution layers. The ECA module achieves the best performance when $k_{size}=3$ and $k_{size}=7$. Furthermore, note that the adaptive approach does not outperform fixed ones. We conjecture the main reason is that one layer of convolution with small filter size is sufficient to capture enough spatial feature information within one channel, and larger perception filter and more layers are redundant [49].

### 5.2. Improvements by Anchor Configuration

Population size ($N_{p}$) and maximum iteration number are two key initialization parameters for the DE solver utilized in the proposed anchor configuration algorithm. To determine the most reasonable initialization parameters, the following experiments were carried out.

**Figure 5:**
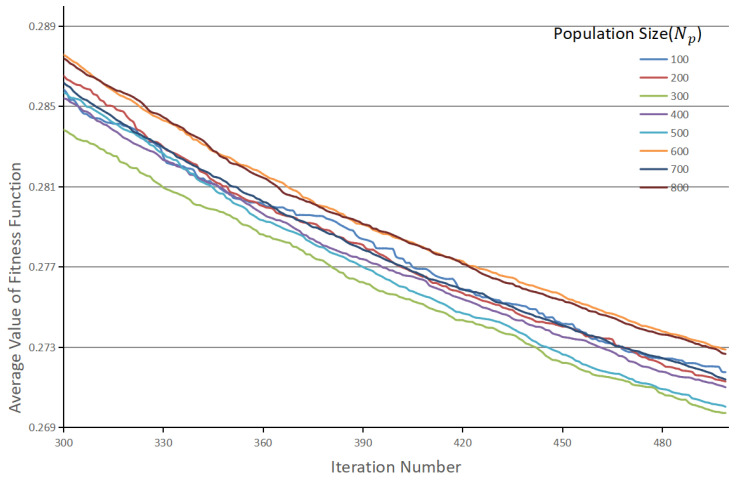
Average value of the fitness function under different population sizes.

Firstly, we set $P_{i}=\theta \in \left(6,2\right)$ for the DE optimizer, in which only six anchors were configured. Therefore, other parameters were set as follows: $C_{r}=0.7$, $F_{s}=0.5$, maximum iteration = 500. The measured average value of the fitness function under different population sizes are listed in Figure 5 and the detailed convergence time are reported in Table 6.

**Figure 6:**
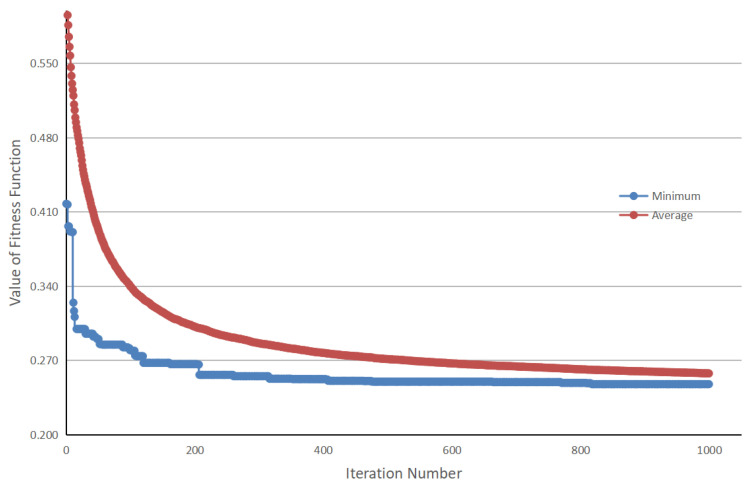
The relationship between average and minimum value of the fitness function.

**Figure 7:**
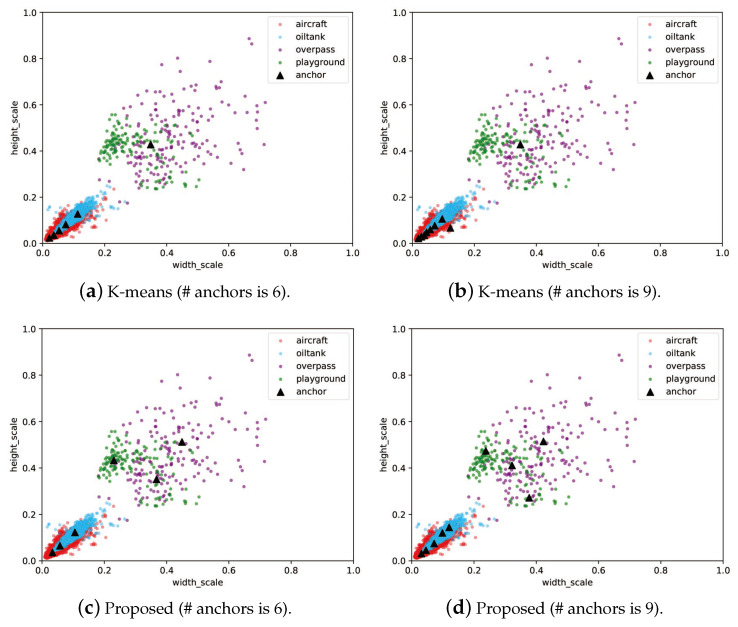
Distributions of the ground truth bounding boxes and the anchors obtained by using K-means clustering and the proposed scheme on the RSOD dataset.

**Figure 8:**
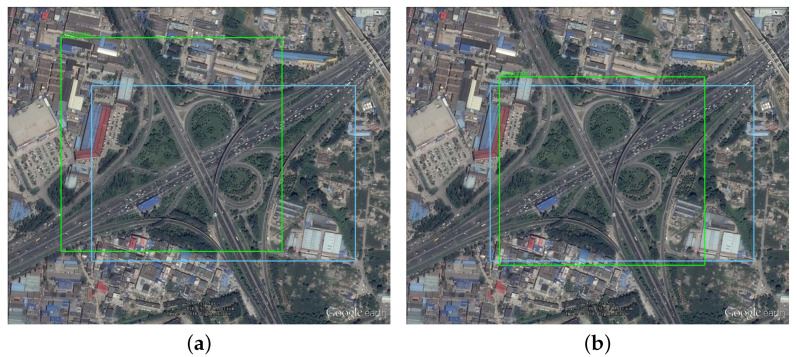
Comparison of detection results of YOLOv4-Tiny with the proposed detector. The ground truth is in blue box, and the predicted target is in green color. (**a**) Original YOLOv4-Tiny network detection results. (**b**) The proposed algorithm detection results.

When $N_{p}$ = 300, the objective function reaches the lowest value among all different configurations, so $N_{p}$ = 300 was selected as the optimal parameter setting. In addition, it was also found that 500 iterations did not make the DE solver fully converge, so the maximum iteration number was increased to 1000. The final performance of DE is shown in Figure 6. As the iteration number increases, the minimum and average values of the fitness function gradually coincide. The best anchor settings obtained for the RSOD dataset are summarized in Table 7. The obtained anchors, except for the fourth one, tends to have a larger scale in the height dimension. This phenomenon reveals that our scheme has optimized the anchor settings to best match the objects at varies scales. To more clearly observe the advantage of our proposed algorithm, we have also visualized the distribution of the ground truth bounding boxes and the obtained anchor boxes by using a scatter plot illustrated in Figure 7, in which the results obtained by using K-means clustering are compared. The figures show that the anchors obtained by the proposed algorithm are more evenly distributed among the entire data set, i.e., anchor allocation fully takes into account the distribution of samples in each category. For instance, in the dimension scale of 0.2 to 0.5, the K-means clustering scheme only allocated a single anchor to capture all the objects with large variant in scales, which will inevitably cause degradation in detection accuracy. In contrast, the proposed scheme has allocated four anchors in this range, each of which also corresponds to the clustering centers of a specific category of object. Therefore, it can be concluded that the proposed algorithm can locate the target objects more accurately than the original YOLOv4-Tiny network as the example shows in Figure 8. Note that, in this experiment, both networks have the same number of prediction layers.

**Figure 9:**
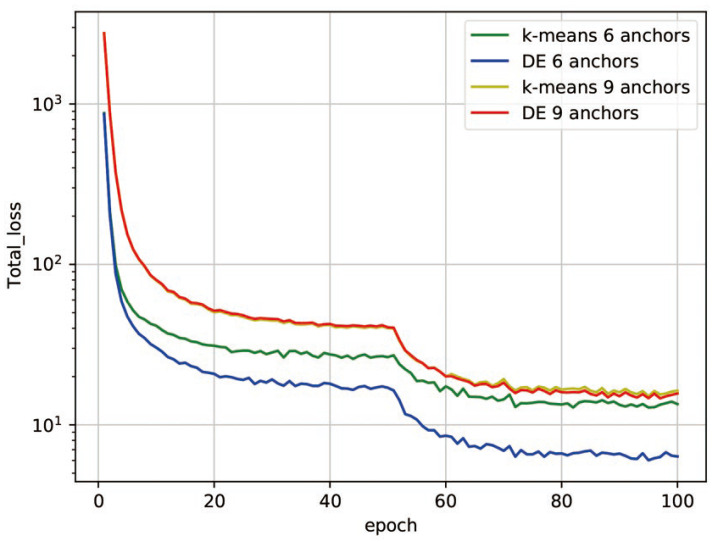
The loss function curve using anchors obtained by different algorithms.

Besides the accuracy improvements, the proposed anchor configuration scheme also delivers a faster training speed over traditional approaches. The loss functions obtained by adopting the aforementioned two anchor selection algorithms in training of the neural network models are compared in Figure 9. From the curves, we can see that the training process which adopted the proposed anchor selection scheme converges more quickly, and the final loss drops by about 50% relative to the K-means clustering scheme in the case of using six anchors. Because anchors selected by the K-means clustering scheme are narrowed in a small region, it is difficult for the detector to capture information from those samples outside this region to achieve better matching results. The anchors obtained by the proposed algorithm distribute more evenly in the dataset, which greatly improves the overall learning efficiency of the neural network.

After obtained the optimal anchor setting, the detection accuracy achieved by adding three prediction layers are shown in Table 8. Compared with K-means clustering, the proposed algorithm can improve the detection accuracy by 1.13% in terms of mAP when using 9 anchors.

**Figure 10:**
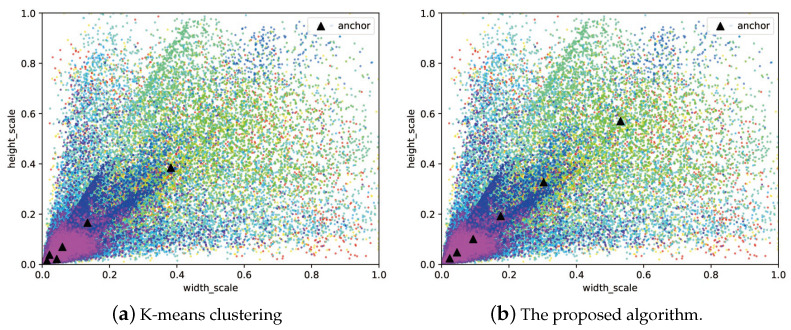
Comparison of the anchor configuration results in the DIOR dataset.

Finally, to verify the generality of the proposed scheme, we have also tested the proposed detection framework on the DIOR dataset. As illustrated in Figure 10, the distribution of the obtained anchors by using the proposed scheme is also more evenly allocated than K-means clustering. More detailed results of the measured AP scores of different categories are compared in Table 9. It can be observed that the proposed scheme outperforms K-means clustering by 4.41% in terms of mAP score.

### 5.3. Comparison with the State-of-the-Art

We first compare the performance of the proposed detector with three generic detectors, including SSD, YOLOv4, and the YOLOv4-Tiny lightweight network on both the RSOD and DIOR datasets, respectively. Table 10 shows the performance of different networks on RSOD. Detection speed was measured on the same desktop GPU. Compared to YOLOv4-Tiny, the proposed scheme can achieve a considerable 5.13% improvement in mAP, while the cost in speed is an ~50 FPS decline on desktop GPUs. However, the differences in speed will become negligible when deployed in embedded platforms. It can also be observed that the proposed network can even achieve a slightly higher accuracy over the SSD model, while the processing speed is 4× faster.

In addition, the proposed model was also trained and evaluated on the DIOR dataset, and the experimental results were reported in Table 11 and Table 12. Compared with YOLOv4-Tiny, our method improved the detection accuracy by 3.58%.

Table 13 compares the proposed scheme with state-of-the-art detectors that have been optimized for remote sensing images. Among all the detectors, CSFF [53] and CF2PN [16] have the highest accuracy but the most complex network structure. For instance, CSFF adopted ResNet-101 as the backbone and an FPN as the prediction network, which greatly improved the accuracy of remote sensing object detection. However, the network model is 8× larger than that of the proposed scheme, resulting in a 15× slower processing speed on desktop GPUs. The extremely large neural network model and high computational workload have prohibited similar schemes like CSFF and CF2PN to be deployed on embedded hardware platforms. In contrast, Simple-CNN [54] and ASSD-lite [55] have used simpler backbone structures and more compact network design. These two methods can then achieve real-time processing speed (around 60 FPS) on desktop GPUs. However, the computational workload of these two detectors is still too large to meet the capacity of our target embedded device (i.e., to achieve a real-time speed of 60 FPS, the network model of the detector should have less than 8 GFLOPs workload). The only lightweight detector that can compete with the proposed scheme in terms of the number of parameters is LO-Det [56]. However, in LO-Det, the authors have designed a very complex FPN network, in which channel shuffle and split operations were repeatedly used in each layer. There operations can improve the network’s accuracy but are very unfriendly to parallel processing on GPUs. Therefore, LO-Det only achieved a 4× slower processing speed than our scheme. The proposed detector achieves a considerably higher processing speed of 227.9 FPS than all the reference schemes on the desktop GPU, which reveals that our scheme is not only lightweight in model structure but also very efficient to be executed on GPU devices for parallel processing.

## 6. Deployment on Embedded Platform

We have deployed the proposed object detection framework on the NVIDIA Jetson Xavier NX board installed on an UAV machine. The proposed lightweight neural network model was quantized into 16-bit floating-point numbers (FP16) by using the TensorRT toolkit. The final prediction accuracy and speed results are reported in Table 14, in which FP32 refers to the standard 32-bit floating-point precision. By quantizing the network model to a reduced precision, the Jetson NX platform can deliver twice the computational capacity than using the standard FP32 data format (i.e., 500 GFLOPs vs. 250 GFLOPs peak performance). In addition, the detection accuracy of the quantized model was well preserved. Thanks to the lightweight, yet efficient network structure proposed, the system can perform high accuracy real-time object detection tasks at the speed of 58.17 FPS on captured remote sensing images with the power consumption of 8.5 W. Therefore, the achieved computational performance is 294 GFLOPs. When compared to the original YOLOv4-Tiny model, our scheme has a significant advantage of 5.1% improvement in detection accuracy, while the sacrifice in speed is not noticeable for practical usage. Moreover, the proposed detector also presented a 15.6% higher computational efficiency over YOLOv4-Tiny. The main reason is that parallelism of the convolutional units is optimized in the device, especially for 3 × 3 conv. Large amount of computation in our proposed is stemming from the increase in 3 × 3 conv, which improves the utilization of device resources.

## 7. Conclusions

In this paper, we have proposed an efficient lightweight object detector for remote sensing images based on deep convolutional neural networks. To achieve the best balance between detection speed and accuracy, we first designed an improved YOLOv4-like backbone network with three prediction layers to alleviate the problem of multi-scale object detection. Further combined with efficient channel attention to obtain important features, the detector can detect small objects with improved accuracy and no significant overhead in computational workload. Then, an optimal anchor configuration scheme was proposed to solve the problem of obtaining biased anchors due to the large variation in object scales in remote sensing images. Finally, evaluation was conducted on both the RSOD and DIOR datasets, respectively, and comparisons with state-of-the-arts show that the proposed lightweight detector has a significant advantage in processing speed while the detection accuracy is maintained at a close level. Furthermore, real-world deployment on the NVIDIA Jetson Xavier NX verified that our scheme was very suitable for low-cost low-power real-time remote sensing object detection tasks.

## Figures and Tables

**Table 1:** Comparison of the performances of the SE and ECA modules. Reduction ratios are set to 16 and 3 for SE and ECA, receptively. CSPA*n* represents the *n*-th CSPA block.

Method	Params/Bytes				mAP/%
	CSPA1	CSPA2	CSPA3	Total	
None	0	0	0	0	80.02
SE	128	512	2048	2688	83.12
ECA	96	192	384	672	83.34

**Table 2:** Characteristics of two different hardware platforms.

Device	NVIDIA GeForce RTX2080Ti Desktop GPU	NVIDIA Jeteson Xavier
GPU	4352 NVIDIA CUDA cores	384-core NVIDIA Volta GPU and 48 Tensorc cores
CPU	Intel Core i9-7960x	6-core NVIDIA Carmel ARM^®^v8.2 64-bit CPU 6 MB L2 + 4 MB L3
Memory	11 GB 352-bit GDDR6 616 GB/s	8 GB 128-bit LPDDR4 51.2 GB/s
Storage	4T Hard Disk Drive	microSD
Power	285 W	10 W (low-power mode)/15 W

**Table 3:** Comparison of model size and accuracy under different number of prediction layers (Num.)

Num.	Params	FLOPs	mAP/%	FPS	Aircraft	Oiltank	Overpass	Playground
2	5.881 M	3.42 G	80.02	285.3	72.46	97.85	51.56	98.22
3	6.527 M	5.06 G	82.00	230.4	74.73	95.99	59.30	97.98
4	6.732 M	9.83 G	83.09	182.5	78.88	96.84	59.67	96.96

**Table 4:** Comparison of different attention methods.

Methods	Params	FLOPs	mAP/%	FPS
None	0 bytes	0 k	80.02	285.3
CBAM	2988 bytes	422 k	79.94	268.9
CA	5424 bytes	351 k	79.95	273.5
SE	2688 bytes	2.94 k	83.12	277.1
ECA	672 bytes	0.89 k	83.34	280.8

**Table 5:** Results of adding the ECA module to the backbone with various numbers of $k_{size}$.

$k_{\mathrm{size}}$	3	5	7	9	Adaptive
mAP/%	83.34	81.70	82.97	80.75	81.56

**Table 6:** The average convergence time under different population sizes.

Population Size	Average Value of the Fitness Function	Average of Convergence Time (s/Iteration)
100	0.2718	7.99
200	0.2713	15.29
300	0.2697	25.73
400	0.2710	36.37
500	0.2700	46.04
600	0.2729	53.67
700	0.2714	58.69
800	0.2727	59.98

**Table 7:** Best anchors (two prediction-layer setting) obtained by the proposed scheme.

Anchor (θ)	Best Decision Variables	Anchor Settings
$\left(\theta_{1}^{\left(1\right)},\theta_{1}^{\left(2\right)}\right)$	(0.0327,0.0370)	(14,15)
$\left(\theta_{2}^{\left(1\right)},\theta_{2}^{\left(2\right)}\right)$	(0.0573,0.0667)	(24,28)
$\left(\theta_{3}^{\left(1\right)},\theta_{3}^{\left(2\right)}\right)$	(0.1065,0.1235)	(44,51)
$\left(\theta_{4}^{\left(1\right)},\theta_{4}^{\left(2\right)}\right)$	(0.2296,0.4331)	(96,180)
$\left(\theta_{5}^{\left(1\right)},\theta_{5}^{\left(2\right)}\right)$	(0.3669,0.3501)	(153,146)
$\left(\theta_{6}^{\left(1\right)},\theta_{6}^{\left(2\right)}\right)$	(0.4491,0.5125)	(187,213)

**Table 8:** The performance of different anchor settings.

Methods	*K*	mAP(%)	Anchors
K-means	6	81.55	(9,10), (15,15), (22,23), (31,34), (47,53), (145,178)
	9	82.00	(8,9), (12,12), (16,15), (19,20), (24,25), (30,32), (40,44), (51,58), (145,178)
Proposed	6	81.69	(14,15), (24,27), (44,51), (96,180), (153,146), (187,213)
	9	83.13	(13,13), (19,19), (30,31), (41,50), (50,60), (134,171), (99,197), (157,113), (176,214)

**Table 9:** Mode detailed mAP (%) results of ablation experiments on the DIOR dataset.

Methods	C1	C2	C3	C4	C5	C6	C7	C8	C9	C10	C11	C12	C13	C14	C15	C16	C17	C18	C19	C20	mAP/%
K-means	42.06	54.45	71.06	68.67	20.22	71.78	43.83	54.48	47.50	58.18	55.90	47.84	44.90	38.59	50.08	33.87	64.52	34.99	23.67	48.74	48.74
DE	57.98	57.80	71.43	74.94	22.83	72.43	43.73	56.71	49.14	59.38	64.80	51.72	47.06	42.68	54.70	38.08	79.72	37.53	26.69	53.64	53.15

**Table 10:** Measured detection results on the RSOD dataset.

Methods	Size	Params	FLOPs	mAP/%	FPS	Aircraft	Oiltank	Overpass	Playground
SSD300 [8]	300	24.15 M	30.64 G	84.71	54.2	70.12	90.34	78.43	100.00
YOLOv4 [35]	416	63.95 M	29.89 G	92.50	44.8	96.13	98.38	75.78	99.71
YOLOv4-Tiny [35]	416	5.881 M	3.42 G	80.02	285.3	72.46	97.85	51.56	98.22
Proposed	416	6.527 M	5.06 G	85.13	227.9	87.10	98.97	56.58	97.86

**Table 11:** The explanation of each category in DIOR.

C1	C2	C3	C4	C5	C6	C7	C8	C9	C10
Airplane	Airport	Baseball field	Basketball court	Bridge	Chimney	Dam	Expressway service area	Expressway toll station	Golf course
**C11**	**C12**	**C13**	**C14**	**C15**	**C16**	**C17**	**C18**	**C19**	**C20**
Ground track field	Harbor	Overpass	Ship	Stadium	Storage tank	Tennis court	Train station	Vehicle	Wind mill

**Table 12:** Measured detection results on the DIOR dataset.

Methods	C1	C2	C3	C4	C5	C6	C7	C8	C9	C10	C11	C12	C13	C14	C15	C16	C17	C18	C19	C20	mAP
YOLOv4-Tiny [35]	58.61	55.99	71.57	74.52	22.19	72.11	47.26	54.83	48.50	60.11	64.46	51.09	46.92	41.93	55.42	37.18	79.78	36.27	26.49	52.23	52.87
Proposed	58.16	55.62	72.39	76.01	25.86	73.03	43.31	55.43	51.39	58.94	66.03	51.30	48.69	70.41	51.82	53.34	82.46	38.78	32.60	63.33	56.45

**Table 13:** Comparison with state-of-the-art detectors for remote sensing images. The performance data were measured on the DIOR dataset.

Approach	CSFF [53]	CF2PN [16]	Simple-CNN [54]	ASSD-Lite [55]	LO-Det [56]	Proposed
Year	2021	2021	2021	2021	2021	2021
Backbone	ResNet-101	VGG16	VGG16	MobileNetv2	MobileNetv2	17-layer-CNN
Parameters	>46 M	91.6 M	23.53 M	>24 M	6.93 M	6.5 M
FPS	15.21	19.7	13	35	64.52	227.9
mAP	68	67.25	66.5	63.3	58.73	56.45
Device	RTX3090	RTX2080Ti	GT710	GTX 1080Ti	RTX3090	RTX2080Ti

**Table 14:** Measured accuracy, speed, and efficiency on the NVIDIA Jetson Xavier NX board.

Methods	mAP (FP32)	mAP (FP16)	FPS (FP16)	Efficiency (100%)
YOLOv4-Tiny	80.02%	80.22%	63.28	43.2%
Proposed	85.13%	85.33%	58.17	58.8%

## Data Availability

The datasets presented in this study are available through: https://onedrive.hyper.ai/home/RSOD-Dataset (accessed on 10 April 2021), http://www.escience.cn/people/gongcheng/DIOR.html (accessed on 10 April 2021).

## References

[1] Khan M.J., Khan H.S., Yousaf A., Khurshid K., Abbas A. (2018). Super-Resolution Modern Trends in Hyperspectral Image Analysis: A Review. IEEE Access.

[2] Hong D.F., Gao L.R., Yokoya N., Yao J., Chanussot J., Du Q., Zhang B. (2021). More Diverse Means Better: Multimodal Deep Learning Meets Remote-Sensing Imagery Classification. IEEE Trans. Geosci. Remote Sens..

[3] Chen J., Wang G.B., Luo L.B., Gong W.P., Cheng Z. (2021). Building Area Estimation in Drone Aerial Images Based on Mask R-CNN. IEEE Geosci. Remote Sens. Lett..

[4] Afaq Y., Manocha A. (2021). Analysis on change detection techniques for remote sensing applications: A review. Ecol. Inform..

[5] Hoeser T., Bachofer F., Kuenzer C. (2020). Object Detection and Image Segmentation with Deep Learning on Earth Observation Data: A Review-Part II: Applications. Remote Sens..

[6] Wang P., Wang L.G., Leung H., Zhang G. (2021). Super-Resolution Mapping Based on SpatialSpectral Correlation for Spectral Imagery. IEEE Trans. Geosci. Remote Sens..

[7] Zhao Z.Q., Zheng P., Xu S.T., Wu X. (2019). Object Detection with Deep Learning: A Review. IEEE Trans. Neural Netw. Learn. Syst..

[8] Liu W., Anguelov D., Erhan D., Szegedy C., Reed S., Fu C.Y., Berg A.C. (2016). SSD: Single Shot MultiBox Detector. European Conference on Computer Vision.

[9] Redmon J., Divvala S., Girshick R., Farhadi A. You only look once: Unified, real-time object detection. Proceedings of the IEEE Conference on Computer Vision and Pattern Recognition.

[10] Zhao Q., Sheng T., Wang Y., Tang Z., Ling H. M2det: A single-shot object detector based on multi-level feature pyramid network. Proceedings of the AAAI Conference on Artificial Intelligence.

[11] Lin T.Y., Dollar P., Girshick R., He K., Hariharan B., Belongie S. Feature Pyramid Networks for Object Detection. Proceedings of the IEEE Conference on Computer Vision and Pattern Recognition.

[12] Ren S., He K., Girshick R., Sun J. Faster R-CNN: Towards Real-Time Object Detection with Region Proposal Networks. Proceedings of the Advances in Neural Information Processing Systems 28 (NIPS 2015).

[13] Du P.J., Xia J.S., Zhang W., Tan K., Liu Y., Liu S.C. (2012). Multiple Classifier System for Remote Sensing Image Classification: A Review. Sensors.

[14] Tsagkatakis G., Aidini A., Fotiadou K., Giannopoulos M., Pentari A., Tsakalides P. (2019). Survey of Deep-Learning Approaches for Remote Sensing Observation Enhancement. Sensors.

[15] Wang J.Y., Wang Y.Z., Wu Y.L., Zhang K., Wang Q. (2020). FRPNet: A Feature-Reflowing Pyramid Network for Object Detection of Remote Sensing Images. IEEE Geosci. Remote Sens. Lett..

[16] Huang W., Li G., Chen Q., Ju M., Qu J. (2021). CF2PN: A Cross-Scale Feature Fusion Pyramid Network Based Remote Sensing Target Detection. Remote Sens..

[17] Chen L.C., Liu C.S., Chang F.L., Li S., Nie Z.Y. (2021). Multiscale object detection in high-resolution remote sensing images via rotation invariant deep features driven by channel attention. Int. J. Remote Sens..

[18] Qing Y.H., Liu W.Y., Feng L.Y., Gao W.J. (2021). Improved YOLO Network for Free-Angle Remote Sensing Target Detection. Remote Sens..

[19] Li X.G., Li Z.X., Lv S.S., Cao J., Pan M., Ma Q., Yu H.B. (2021). Ship detection of optical remote sensing image in multiple scenes. Int. J. Remote Sens..

[20] Chen L.C., Liu C.S., Chang F.L., Li S., Nie Z.Y. (2021). Adaptive multi-level feature fusion and attention-based network for arbitrary-oriented object detection in remote sensing imagery. Neurocomputing.

[21] Tian Z., Zhan R., Hu J., Wang W., He Z., Zhuang Z. (2020). Generating Anchor Boxes Based on Attention Mechanism for Object Detection in Remote Sensing Images. Remote Sens..

[22] Mo N., Yan L., Zhu R., Xie H. (2019). Class-Specific Anchor Based and Context-Guided Multi-Class Object Detection in High Resolution Remote Sensing Imagery with a Convolutional Neural Network. Remote Sens..

[23] Long Y., Gong Y., Xiao Z., Liu Q. (2017). Accurate object localization in remote sensing images based on convolutional neural networks. IEEE Trans. Geosci. Remote Sens..

[24] Mhangara P., Mapurisa W. (2019). Multi-Mission Earth Observation Data Processing System. Sensors.

[25] Han W.Y., Liu X.H. Clustering Anchor for Faster R-CNN to Improve Detection Results. Proceedings of the IEEE International Conference on Artificial Intelligence and Computer Applications (ICAICA).

[26] Chen L., Zhou L., Liu J. (2020). Aircraft Recognition from Remote Sensing Images Based on Machine Vision. J. Inf. Process. Syst..

[27] Redmon J., Farhadi A. YOLO9000: Better, faster, stronger. Proceedings of the IEEE Conference on Computer Vision and Pattern Recognition.

[28] Al-Naji A., Perera A., Mohammed S.L., Chahl J. (2019). Life Signs Detector Using a Drone in Disaster Zones. Remote Sens..

[29] Nikulin A., de Smet T.S., Baur J., Frazer W.D., Abramowitz J.C. (2018). Detection and Identification of Remnant PFM-1 ’Butterfly Mines’ with a UAV-Based Thermal-Imaging Protocol. Remote Sens..

[30] NVIDIA Developer NVIDIA Embedded-Computing. https://developer.nvidia.com/embedded-computing.

[31] Girshick R., Donahue J., Darrell T., Malik J. Rich Feature Hierarchies for Accurate Object Detection and Semantic Segmentation. Proceedings of the IEEE Conference on Computer Vision and Pattern Recognition.

[32] Girshick R. Fast r-cnn. Proceedings of the IEEE Conference on Computer Vision and Pattern Recognition.

[33] Tian Z., Shen C., Chen H., He T. (2019). FCOS: Fully Convolutional One-Stage Object Detection. arXiv.

[34] Joseph R., Ali F. (2018). YOLOv3: An Incremental Improvement. arXiv.

[35] Bochkovskiy A., Wang C., Liao H. (2020). YOLOv4: Optimal Speed and Accuracy of Object Detection. arXiv.

[36] Gao J.F., Chen Y., Wei Y.M., Li J.N. (2021). Detection of Specific Building in Remote Sensing Images Using a Novel YOLO-S-CIOU Model. Case: Gas Station Identification. Sensors.

[37] Hu X.L., Liu Y., Zhao Z.X., Liu J.T., Yang X.T., Sun C.H., Chen S.H., Li B., Zhou C. (2021). Real-time detection of uneaten feed pellets in underwater images for aquaculture using an improved YOLO-V4 network. Comput. Electron. Agric..

[38] Singh S., Ahuja U., Kumar M., Kumar K., Sachdeva M. (2021). Face mask detection using YOLOv3 and faster R-CNN models: COVID-19 environment. Multimed. Tools Appl..

[39] He K., Zhang X., Ren S., Sun J. Deep residual learning for image recognition. Proceedings of the IEEE Conference on Computer Vision and Pattern Recognition.

[40] Simonyan K., Zisserman A. (2014). Very Deep Convolutional Networks for Large-Scale Image Recognition. arXiv.

[41] Sandler M., Howard A., Zhu M., Zhmoginov A., Chen L.C. Mobilenetv2: Inverted residuals and linear bottlenecks. Proceedings of the IEEE Conference on Computer Vision and Pattern Recognition.

[42] Lecun Y., Bengio Y., Hinton G. (2015). Deep learning. Nature.

[43] Wang F., Jiang M., Qian C., Yang S., Li C., Zhang H., Wang X., Tang X. Residual attention network for image classification. Proceedings of the IEEE Conference on Computer Vision and Pattern Recognition.

[44] Hu J., Shen L., Albanie S., Sun G., Wu E. Squeeze-and-Excitation Networks. Proceedings of the IEEE Conference on Computer Vision and Pattern Recognition.

[45] Woo S., Park J., Lee J.Y., Kweon I.S. CBAM: Convolutional Block Attention Module. Proceedings of the European Conference on Computer Vision (ECCV).

[46] Hou Q., Zhou D., Feng J. (2021). Coordinate Attention for Efficient Mobile Network Design. arXiv.

[47] Junos M.H., Khairuddin A., Thannirmalai S., Dahari M. (2021). An optimized YOLO-based object detection model for crop harvesting system. IET Image Process..

[48] Zlocha M., Dou Q., Glocker B. Improving RetinaNet for CT Lesion Detection with Dense Masks from Weak RECIST Labels. Proceedings of the Medical Image Computing and Computer-Assisted Intervention (MICCAI).

[49] Wang Q., Wu B., Zhu P., Li P., Hu Q. ECA-Net: Efficient Channel Attention for Deep Convolutional Neural Networks. Proceedings of the IEEE Conference on Computer Vision and Pattern Recognition.

[50] Price K.V. Differential evolution: A fast and simple numerical optimizer. Proceedings of the North American Fuzzy Information Processing.

[51] Opara K.R., Arabas J. (2019). Differential Evolution: A survey of theoretical analyses. Swarm Evol. Comput..

[52] Li K., Wan G., Cheng G., Meng L., Han J. (2020). Object detection in optical remote sensing images: A survey and a new benchmark. ISPRS J. Photogramm. Remote Sens..

[53] Cheng G., Si Y.J., Hong H.L., Yao X.W., Guo L. (2021). Cross-Scale Feature Fusion for Object Detection in Optical Remote Sensing Images. IEEE Geosci. Remote Sens. Lett..

[54] Li L., Cao G., Liu J., Tong Y. (2021). Efficient Detection in Aerial Images for Resource-Limited Satellites. IEEE Geosci. Remote Sens. Lett..

[55] Xu T., Sun X., Diao W.H., Zhao L.J., Fu K., Wang H.Q. (2021). ASSD: Feature Aligned Single-Shot Detection for Multiscale Objects in Aerial Imagery. IEEE Trans. Geosci. Remote Sens..

[56] Huang Z., Li W., Xia X.G., Wang H., Tao R. (2021). LO-Det: Lightweight Oriented Object Detection in Remote Sensing Images. IEEE Trans. Geosci. Remote Sens..

